# Supplementary material for: Active site specificity profiling datasets of matrix metalloproteinases (MMPs) 1, 2, 3, 7, 8, 9, 12, 13 and 14
Source: Data Brief. 2016 Feb 22;7:299–310. doi: 10.1016/j.dib.2016.02.036 (PMC4777984; doi:10.1016/j.dib.2016.02.036)
Supplement: Supplementary file 10 — Supplementary material [file mmc10.zip › WebPICS_hMMP3_G_1%/P2prime.html]

 

PICS results


|  |  |
| --- | --- |
| **P2prime\_C**  3 in 151 sites   2.0 %    effects > 10 perc. pnts.  (vice-versa in brackets)  P2\_C: 62.1 (26.6)   P1\_Y: 62.1 (26.6) |  |
| **P2prime\_H**  5 in 151 sites   3.3 %    effects > 10 perc. pnts.  (vice-versa in brackets)  P3\_I: 31.4 (12.1)   P3\_L: 48.7 (14.3)   P2\_C: 55.4 (39.6)   P1prime\_I: 62.8 (12.1)   P3prime\_A: 62.8 (12.1) |  |
| **P2prime\_I**  18 in 151 sites   11.9 %    effects > 10 perc. pnts.  (vice-versa in brackets)  P2\_F: 12.7 (38.1)   P2\_L: 16.5 (17.5)   P1\_D: 23.4 (28.1)   P1\_Q: 19.2 (26.6)   P1prime\_C: 10.1 (18.1)   P1prime\_V: 19.4 (16.7)   P3prime\_N: 26.0 (42.6) |  |
| **P2prime\_K**  24 in 151 sites   15.9 %    effects > 10 perc. pnts.  (vice-versa in brackets)  P2\_S: 12.2 (22.6) |  |
| **P2prime\_L**  19 in 151 sites   12.6 %    effects > 10 perc. pnts.  (vice-versa in brackets)  P3\_A: 28.9 (27.4)   P1\_K: 20.3 (43.0)   P1\_S: 17.0 (23.1)   P1prime\_I: 19.6 (14.3)   P3prime\_G: 13.8 (23.8)   P3prime\_K: 15.0 (11.4) |  |
| **P2prime\_Q**  10 in 151 sites   6.6 %    effects > 10 perc. pnts.  (vice-versa in brackets)  P3\_L: 18.7 (11.0)   P1\_H: 16.7 (33.4)   P3prime\_L: 28.7 (16.9) |  |
| **P2prime\_R**  13 in 151 sites   8.6 %    effects > 10 perc. pnts.  (vice-versa in brackets)  P3\_I: 14.5 (14.5)   P2\_H: 19.1 (41.4)   P1\_S: 13.8 (12.8) |  |
| **P2prime\_T**  10 in 151 sites   6.6 %    effects > 10 perc. pnts.  (vice-versa in brackets)  P3\_I: 21.4 (16.5)   P2\_H: 16.0 (26.7)   P2\_L: 28.7 (16.9)   P1prime\_V: 26.1 (12.4)   P3prime\_A: 32.8 (12.6)   P3prime\_I: 12.1 (10.1) |  |
| **P2prime\_V**  20 in 151 sites   13.2 %    effects > 10 perc. pnts.  (vice-versa in brackets)  P1\_F: 16.0 (53.5)   P3prime\_I: 17.1 (28.5) |  |
